# Impact of a district mental health care plan on suicidality among patients with depression and alcohol use disorder in Nepal

**DOI:** 10.1371/journal.pone.0231158

**Published:** 2020-04-07

**Authors:** Luke R. Aldridge, Emily C. Garman, Nagendra P. Luitel, Mark J. D. Jordans

**Affiliations:** 1 Johns Hopkins Bloomberg School of Public Health, Baltimore, Maryland, United States of America; 2 Alan J F Alan J Flisher Centre for Public Mental Health, Department of Psychiatry and Mental Health, University of Cape Town, Cape Town, South Africa; 3 Transcultural Psychosocial Organization Nepal, Kathmandu, Nepal; 4 Centre for Global Mental Health, Health Service and Population Research Department, Institute of Psychiatry, Psychology and Neuroscience, King’s College London, London, United Kingdom; Chinese Academy of Medical Sciences and Peking Union Medical College, CHINA

## Abstract

**Background:**

Large scale efforts to expand access to mental healthcare in low- and middle-income countries have focused on integrating mental health services into primary care settings using a task sharing approach delivered by non-specialist health workers. Given the link between mental disorders and risk of suicide mortality, treating common mental disorders using this approach may be a key strategy to reducing suicidality.

**Methods and findings:**

The Programme for Improving Mental Health Care (PRIME) evaluated mental health services for common mental disorders delivered by non-specialist health workers at ten primary care facilities in Chitwan, Nepal from 2014 to 2016. In this paper, we present the indirect impact of treatment on suicidality, as measured by suicidal ideation, among treatment and comparison cohorts for depression and AUD using multilevel logistic regression. Patients in the treatment cohort for depression had a greater reduction in ideation relative to those in the comparison cohort from baseline to three months (OR = 0.16, 95% CI: 0.05–0.59; p = 0.01) and twelve months (OR = 0.31, 95% CI: 0.08–1.12; p = 0.07), with a significant effect of treatment over time (p = 0.02). Among the AUD cohorts, there were no significant differences between treatment and comparison cohorts in the change in ideation from baseline to three months (OR = 0.64, 95% CI: 0.07–6.26; p = 0.70) or twelve months (OR = 0.46, 95% CI: 0.06–3.27; p = 0.44), and there was no effect of treatment over time (p = 0.72).

**Conclusion:**

The results provide evidence integrated mental health services for depression benefit patients by accelerating the rate at which suicidal ideation naturally abates over time. Integrated services do not appear to impact ideation among people with AUD, though baseline levels of ideation were much lower than for those with depression and may have led to floor effects. The findings highlight the importance of addressing suicidality as a specific target–rather than an indirect effect–of treatment in community-based mental healthcare programs.

## Introduction

More than 800,000 people die by suicide each year, with three quarters of suicides occurring in low- and middle-income countries (LMIC) [1] where scarce resources exist for mental health care and suicide prevention efforts [2]. The burden of suicide is also increasingly shifting to Asia given population growth in this region and declining suicide rates in high-income Western countries [3]. The 11 countries in the World Health Organization (WHO) South-East Asia Region comprises approximately one quarter of the world’s population, yet account for 39% all suicides [1]. In Nepal, civil unrest, natural disaster, and economic strife have contributed to rising suicide rates, which is currently a leading cause of mortality among women of reproductive age [4,5]. Compounding the issue, countries in this region lack adequate health surveillance systems to enumerate suicide rates within their populations [6]. As a result, available estimates likely underreport the burden of suicide, a finding evidenced by discrepancies between estimated mortality rates produced by government reports and rigorous research studies [6].

There has been growing recognition of the need to address suicide as an issue in recent years. Through the Sustainable Development Goals, the United Nations has set a goal by 2030 to reduce mortality from non-communicable diseases by one third through prevention and treatment and the promotion of mental health and well-being [7]. Achieving this target is evaluated using two indicators: suicide mortality and mortality from non-communicable diseases [8]. The WHO has also highlighted the importance of addressing the burden of suicide through the Comprehensive Mental Health Action Plan, which calls for reducing mortality from suicide by 10% from 2012 to 2020 [9].

Currently, less than one in three people who demonstrate suicidal behaviors (defined as suicidal thoughts, plans, or attempts) receive treatment in middle-income countries, and only one in six in low-income countries [10]. Some population-level prevention efforts in (LMIC) have proven effective in reducing mortality, such as pesticide control to reduce access to means of suicide [11,12]. At the individual level, expanding access to mental health care could be a key strategy to reducing suicide given the link between mental and substance use disorders and suicidality [13–17]. Between 80% and 98% of people who attempt or complete suicide have a mental or substance use disorder [14,16], and people with these disorders are at least ten times more likely to attempt or complete suicide than the general population [13]. In particular, people with depression, substance use disorders, and psychosis are at greatest risk of suicidal behavior [13]. Expanding access to mental healthcare can address mental and substance use disorders underlying increased risk of suicidality. Recent large-scale efforts to implement and expand access to community-based mental healthcare have focused on integrating mental health services into primary care, with services delivered by non-specialist primary care workers (PCWs) [18–20]. The WHO established this approach a predominant strategy to reducing the mental health treatment gap in the Mental Health Gap Action Programme (mhGAP), which outlines the implementation and scale up of integrated mental healthcare using a supervised, stepped care model [21].

The Programme for Improving Mental Health Care (PRIME) implemented mhGAP to address priority mental health disorders within a district mental health care plan between September 2014 and July 2016 in Chitwan, Nepal. The plan included twelve intervention packages at the community, health facility and organizational level, including community awareness building, stakeholder engagement, and advocacy [22]. At the health facility level, non-specialist PCWs provided services for four priority mental disorders–alcohol use disorder (AUD), depression, epilepsy, and psychosis– according to clinical decision-making guidelines in disorder-specific modules of the mhGAP Intervention Guide (mhGAP-IG) [22–24]. Patients receiving services were screened and referred for serious risk of suicide or self-half according to the mhGAP-IG, though notably, the mhGAP module on suicide was not included within PRIME services. A cohort study evaluating the individual-level impact of PRIME integrated mental health services demonstrated small to moderate treatment effects on clinical symptoms and daily functioning for patients with depression, AUD, and psychosis; treatment did not appear to have a significant impact on either for patients with epilepsy [25].

While increasing access to mental health care should theoretically reduce suicidality given the link between suicide and mental disorders, the indirect impact of non-specialist care for depression and AUD on suicidality has yet to be evaluated at scale. At present, we evaluate the impact of mental health services delivered by non-specialist PCWs on suicidal ideation among patients in the PRIME Nepal cohort with depression and AUD, and consider differences in outcomes across relevant sociodemographic subgroups.

## Methods

### Setting and participants

PRIME implemented and evaluated district-level mental health care packages in Ethiopia, India, Nepal, South Africa and Uganda. The cohort study protocol [26], major challenges to implementation [27], pilot testing [22], and program impact [25] in Nepal are presented elsewhere. In brief, mental health services delivered by non-specialist PCWs were implemented at ten primary health facilities in Chitwan, a predominantly rural district on the central southern border with India. At the time of study, Chitwan had approximately 580,000 residents and a literacy rate of 79%, which is greater than the national average of 68% [28]. Previous research in Chitwan found 11% and 5% of adults screened positive for AUD and depression, respectively [29], while treatment seeking among those who screen positive for either disorder was less than 2% [30]. Within PRIME, PCWs were trained in the detection, assessment, and treatment provision for priority mental disorders. Health workers provided treatment following guidelines for clinical decision-making set forth by a module for each disorder in the mhGAP-IG [24], which included a combination of pharmacological and psychosocial interventions, including psychotropic medication, psychoeducation, brief emotional support, and behavioral and motivational activation. Prior to PRIME, mental health services were only available at the district hospital and a private medical college.

Participants were eligible to enroll in a cohort if they were above the age of majority (≥16 years), resided in Chitwan, were fluent in the local language, and willing and capable of providing informed consent. People who were already receiving treatment for depression or AUD during recruitment were not eligible for inclusion.

### Study design and recruitment

The primary objective of the study is to evaluate the indirect effect of treatment for depression and AUD on suicidality (i.e., suicidal thoughts, plans, and attempts) among people who receive non-specialist services within PRIME Nepal. PRIME field workers approached individuals attending ten study health facilities in Chitwan for recruitment and informed consent. Field workers also used proactive community case detection with community informants to identify individuals who may be eligible for and benefit from study participation [31]. Eligible patients who agreed to participate were then screened for depression or AUD by PRIME field workers prior to their consultation with a primary health care worker. Field workers used the Patient Health Questionnaire (PHQ-9) [32] to screen for depression and the Alcohol Use Disorder Identification Test (AUDIT) [33] to screen for AUD. If a participant screened positive, she or he would complete a psychiatric diagnostic interview by a trained primary health care worker or medical officer. Those who subsequently received a confirmatory diagnosis for depression or AUD during diagnostic interview were enrolled in the respective cohort, with priority given to AUD when both disorders were diagnosed. Participants who screened positive but failed to receive a confirmatory diagnosis during the diagnostic interview were enrolled into two comparison cohorts, one for depression and one for AUD. The comparison cohorts received standard primary care at the health facilities consisting of assessment, diagnosis, and treatment of somatic conditions, with no mental health treatment provided.

Participants enrolled in the treatment cohorts received psychotropic medication, psychosocial support, or psychoeducation according to the clinical decision algorithm in the mhGAP Intervention Guide [24]. Half of participants in each treatment cohort were also randomly assigned to receive a psychological intervention, the Healthy Activity Program (HAP) for depression and the Counseling for Alcohol Problems (CAP) for AUD, as part of a nested randomized control trial [34]. HAP is a behavioral activation therapy delivered individually over six to eight weekly sessions and includes psychoeducation, activity structuring and monitoring, problem solving, and activating social networks [35]. CAP is a manualized motivational activation delivered individually over four weekly sessions, consisting of personal appraisal, development cognitive and behavioral skills related to alcohol use, and management of potential relapse [36]. Health facilities in the study received organizational support to provide mental health services in the form of an ensured supply of psychotropic medication and mechanisms for monitoring, capacity building, and resource mobilization.

### Measures

The primary outcome, recent suicidal ideation, was assessed through participants responding to the prompt, “Have you thought of taking your life in the past three months?” via text questionnaire on electronic tablet. Information was also gathered on past suicide attempts, however, ideation was selected as the primary outcome since the present study is not sufficiently powered to detect changes in suicide attempts or completions. To measure depression, participants reported the frequency of depressive symptoms within the previous two weeks in a Likert-scale response to nine items using the PHQ-9, each item response ranging from 0 (“not at all”) to three (“nearly every day”) [32]. A validation study of the PHQ-9 in Nepal indicates an internal consistency of α = 0.84, with a total score of 10 or above suggesting high risk for depression at 94% sensitivity and 80% specificity [37]. Participants reported symptoms related to harmful alcohol use using the ten-item AUDIT, with each item ranging from 0 to 4. The AUDIT was developed by the WHO and has been used widely in LMIC for the measurement and detection of AUD [33]. Validation research in Nepal indicates the instrument has an internal consistency of α = 0.82 [38]. The same study also indicated a cut-off of eight or above has a sensitivity for detecting alcohol abuse or dependence of 97.2% and 97.5% for females and males, respectively, and a specificity of 86.7% and 87.8% for females and males, respectively [38]. All participants completed self-report measures at baseline and three- and twelve-month follow up. Sociodemographic information was also collected at baseline through self-report questionnaire.

### Analysis

We used multilevel logistic regression of longitudinal data with participant and primary health facility random effects to evaluate the difference in odds of suicidal ideation over time and across treatment and comparison cohorts for depression and AUD, separately. A treatment-by-time-point interaction term in each model assessed the impact of treatment over time. Sociodemographic factors significantly associated with ideation in a univariable model were considered for inclusion in the final regression model. Missing outcome data (15%) were assumed to be missing at random and were multiply imputed.

### Ethics

Ethical approval was obtained from the Nepal Health Research Council; the Faculty of Health Sciences, University of Cape Town, South Africa; and the WHO, Geneva, Switzerland.

## Results

A total of 2,044 people were eligible and consented to take part in the cohort studies. Of these, 137 received a primary diagnosis of depression and enrolled into the treatment cohort, while 72 were enrolled into the depression comparison cohort. Similarly, 175 received a primary diagnosis of AUD and enrolled in treatment, while 57 were enrolled into the AUD comparison cohort. Baseline sociodemographic characteristics are presented in Table 1. Participants in the depression cohorts were predominantly female (treatment: 83.1%, comparison: 87.5%), over the age of 35 (treatment: 59.1%, comparison: 57.0%), married (treatment: 81.0%, comparison: 88.9%%), and Hindu (treatment: 86.1%, comparison: 80.6%) and had completed less than primary education (treatment: 55.4%, comparison: 51.4%). Participants in the AUD cohorts were predominantly male (treatment: 85.1%, comparison: 91.2%), over the age of 35 (treatment 70.8%, comparison: 78.9%), married (treatment: 94.3%, comparison: 87.7%), and Hindu (treatment: 76.6%, comparison: 84.2%) and had completed primary school or above (treatment: 60.0%, comparison: 54.4%).

**Table 1:** Baseline characteristics of the study population by treatment cohort and primary diagnosis.

	Depression n (%)			Alcohol Use Disorder n (%)		
	Treatment (n = 137)	Comparison (n = 72)	p-value*	Treatment (n = 175)	Comparison n = (57)	p-value*
Baseline ideation	65 (47.5%)	31 (43.1%)	0.55	37 (21.1%)	9 (15.8%)	0.38
Male sex	19 (13.9%)	9 (12.5%)	0.78	149 (85.1%)	52 (91.2%)	0.22
Age group (years)			0.21			0.01
≥25	19 (13.9%)	16 (22.2%)		4 (2.3%)	2 (3.5%)	
26–35	37 (27.0%)	15 (20.8%)		47 (26.9%)	10 (17.5%)	
36–50	45 (32.8%)	28 (38.9%)		80 (45.7%)	24 (42.1%)	
>50	36 (26.3%)	13 (18.1%)		44 (25.1%)	21 (36.8%)	
Education			0.79			0.20
Uneducated or illiterate	38 (27.7%)	17 (23.6%)		37 (21.1%)	9 (15.8%)	
Non-formal, less than primary	38 (27.7%)	20 (27.8%)		33 (18.9%)	17 (29.8%)	
Primary school and above	61 (44.5%)	35 (48.6%)		105 (60.0%)	31 (54.4%)	
Marital status			0.07			0.20
Single	9 (6.6%)	6 (8.3%)		6 (3.4%)	3 (5.3%)	
Has a partner	111 (81.0%)	64 (88.9%)		165 (94.3%)	50 (87.7%)	
Divorced or widowed	17 (12.4%)	2 (2.8%)		4 (2.3%)	4 (7.0%)	
Caste			0.02			0.05
Brahmin or Chhetri	59 (43.1%)	21 (29.2%)		62 (35.4%)	13 (22.8%)	
Janajati	38 (27.7%)	15 (20.8%)		44 (25.1%)	18 (31.6%)	
Dalit	32 (23.4%)	32 (44.4%)		50 (28.6%)	24 (42.1%)	
Others	8 (5.8%)	4 (5.6%)		19 (10.9%)	2 (3.5%)	
Religion			0.31			0.30
Muslim	0 (0.0%)	0 (0.0%)		1 (0.6%)	0 (0.0%)	
Hindu	118 (86.1%)	58 (80.6%)		134 (76.6%)	48 (84.2%)	
Buddhist	13 (9.5%)	7 (9.7%)		31 (17.7%)	9 (15.8%)	
Christian	6 (4.4%)	7 (9.7%)		9 (5.1%)	0 (0.0%)	

* p-value of χ^2^ test comparing treatment cohort to comparison cohort within a given disorder

### Depression cohort

**Fig 1:**
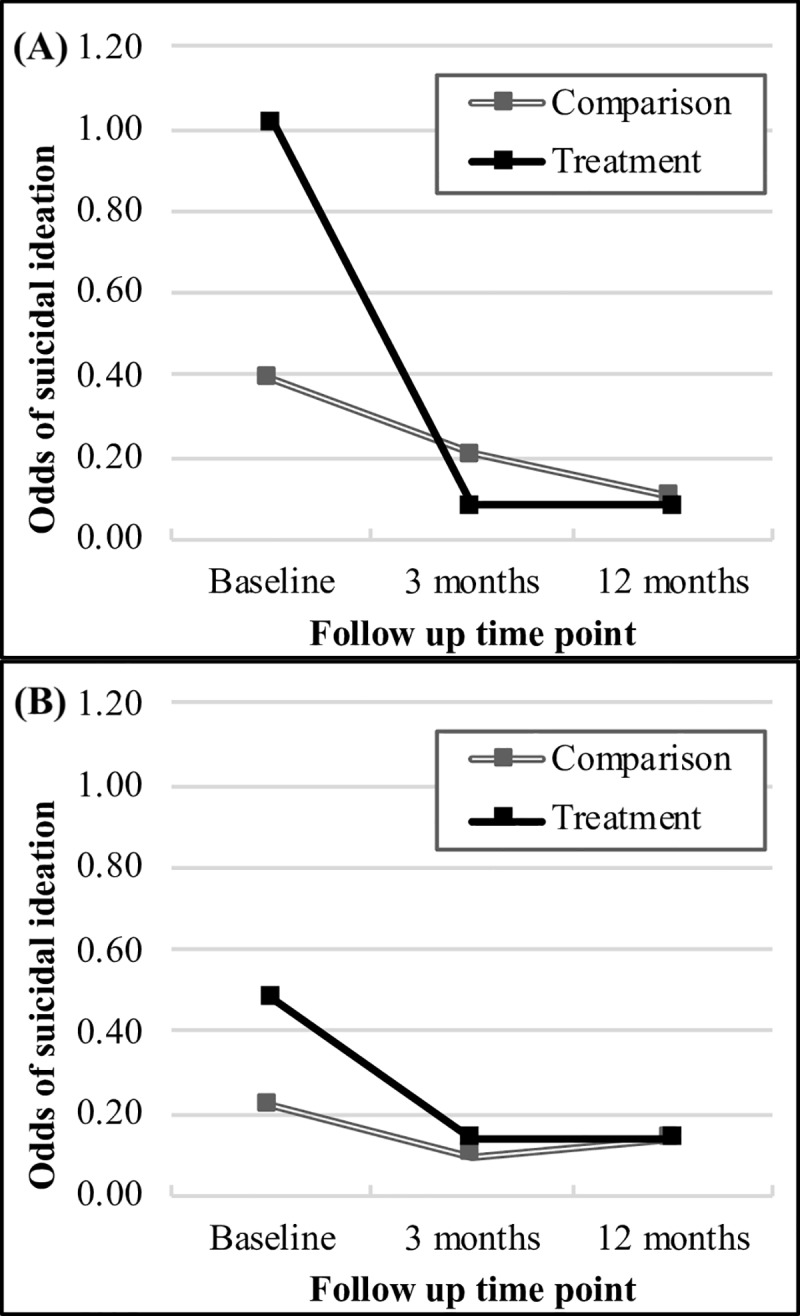
Odds of suicidal ideation for the treatment and comparison cohorts over time. (A) Treatment and comparison cohorts for depression, adjusted for sex and age category. (B) Treatment and comparison cohorts for alcohol use disorder (AUD), adjusted for age category.

At baseline, 47.5% and 43.1% of participants in the depression treatment and comparison cohorts reported suicidal ideation, respectively. In the regression model adjusted for relevant sociodemographic factors, participants in the depression comparison cohort had a 47% reduction in the odds of suicidal ideation from baseline at three months (odds ratio (OR) = 0.53, 95% confidence interval (CI): 0.20–1.38; p = 0.19) and a 73% reduction at 12 months (OR = 0.27, 95% CI: 0.10–0.72; p = 0.01), though the reduction in odds was only significant at 12 months. Adjusted odds of ideation were marginally greater at baseline for those in the treatment cohort compared to the comparison cohort (OR = 2.61, 95% CI: 0.98–6.94; p = 0.06), and were significantly lower at 3 months (OR = 0.23, 95% CI: 0.08–0.66; p<0.01) and twelve months (OR = 0.21, 95% CI: 0.07–0.63; p<0.01) compared to the baseline of the comparison cohort. Odds of ideation for the treatment and comparison cohorts for depression and AUD are depicted in Fig 1.

Those in the treatment cohort had a significantly greater reduction in ideation compared to the comparison cohort over time, with a 84% greater reduction in odds from baseline to three months (OR = 0.16, 95% CI: 0.05–0.59; p = 0.01); however, the confidence interval for the difference between treatment and comparison cohorts from baseline to twelve months crosses the null (OR = 0.31, 95% CI: 0.08–1.12; p = 0.07). Notably, a Wald test of the interaction term between treatment and time point indicates a significant effect of treatment over time when controlling for sociodemographic factors (p = 0.02). A Wald test for follow up also indicates a significant secular effect of time (p = 0.03) There is no significant difference between treatment and comparison cohorts in ideation when holding sociodemographic factors constant and not allowing the treatment effect to differ over time (OR 2.61, 95% CI: 0.98–6.94; p = 0.06). Results of adjusted and unadjusted multilevel logistic regression for the depression cohorts are presented in Table 2.

**Table 2:** Unadjusted and adjusted logistic regression of suicidal ideation on treatment cohort, timepoint, and demographics for depression.

	Unadjusted			Adjusted*		
	Odds ratio for ideation	(95% CI)	P-value	Odds ratio for ideation	(95% CI)	P-value**
**Follow up**			0.13**			0.03**
**Comparison cohort**						
Baseline	Ref.			Ref.		
3 months	0.61	(0.25, 1.45)	0.26	0.53	(0.20, 1.38)	0.19
12 months	0.39	(0.16, 0.96)	0.04	0.27	(0.10, 0.72)	0.01
**Treatment cohort**						
Baseline	1.86	(0.82, 4.22)	0.14	2.61	(0.98, 6.94)	0.06
3 months	0.31	(0.12, 0.80)	0.02	0.23	(0.08, 0.66)	<0.01
12 months	0.32	(0.13, 0.80)	0.02	0.21	(0.07, 0.63)	<0.01
**Cohort**						
Treatment vs comparison	1.86	(0.82, 4.22)	0.14	2.61	(0.98, 6.94)	0.06
**Treatment-by-timepoint interaction**			0.10**			0.02**
Treatment vs. comparison, baseline to 3 months	0.28	(0.09, 0.89)	0.03	0.16	(0.05, 0.59)	0.01
Treatment vs. comparison, baseline to 12 months	0.45	(0.14, 1.41)	0.17	0.31	(0.08, 1.12)	0.07
**Sex**						
Female vs. male	–	–		2.30	(0.75, 6.98)	0.14
**Age group (years)**						0.37**
Less than 26	–	–		Ref.		
26 to 35	–	–		0.76	(0.25, 2.31)	0.25
36 to 50	–	–		0.66	(0.23, 1.88)	0.23
51 or older	–	–		0.36	(0.11, 1.18)	0.11

*Adjusted for sex and age category

** p-value for Wald test of interaction terms or multiple factors (e.g., multiple time points)

Age, caste, education level, marriage status, religion, and sex were considered for inclusion in the regression model for depression after fitting an unadjusted model. Each factor was first regressed in a univariable model with suicidal ideation; factors significantly associated in the univariable model were then considered for inclusion in the model of main effects and retained if found to contribute to model fit. Age and sex were retained in the final adjusted model through this process. After controlling for other variables, there were no differences when comparing odds of ideation among females to males in the depression cohorts (OR = 2.3, 95% CI: 0.75–6.98; p = 0.14), nor was there a significant trend among age categories (p = 0.37).

### AUD cohort

At baseline, 21.1% of participants in the treatment cohort and 15.8% of participants in the comparison cohort reported suicidal ideation. Results of unadjusted and adjusted multilevel logistic regression for the AUD cohorts are presented in Table 3. In a regression model adjusted for relevant sociodemographic factors, odds of suicidal ideation among patients in the comparison cohort were not significantly different at three months (OR = 0.45, 95% CI: 0.05–3.69; p = 0.46) or twelve-months (OR = 0.61, 95% CI: 0.11–3.55; p = 0.59) compared to baseline. Similarly, there were no significant difference in ideation between the comparison cohort at baseline and the treatment cohort at baseline (OR = 2.20, 95% CI: 0.55–8.88; p = 0.27), three months (OR = 0.64, 95% CI: 0.15–2.80; p = 0.55), or twelve months (OR = 0.62, 95% CI: 0.14–2.69; p = 0.52).

**Table 3:** Unadjusted and adjusted logistic regression of suicidal ideation on treatment cohort, timepoint, and demographics for alcohol use disorder.

	Unadjusted			Adjusted*		
	Odds ratio for ideation	(95% CI)	P-value	Odds ratio for ideation	(95% CI)	P-value
**Follow up**			0.90**			0.71**
**Comparison**						
Baseline	Ref.			Ref.		
3 months	0.90	(0.21, 3.81)	0.89	0.45	(0.05, 3.69)	0.46
12 months	0.70	(0.16, 3.04)	0.64	0.61	(0.11, 3.55)	0.59
**Treatment**						
Baseline	2.08	(0.65, 6.63)	0.22	2.20	(0.55, 8.88)	0.27
3 months	0.97	(0.29, 3.25)	0.96	0.64	(0.15, 2.80)	0.55
12 months	0.97	(0.29, 3.31)	0.96	0.62	(0.14, 2.69)	0.52
**Cohort**						
Treatment vs. comparison	2.08	(0.65, 6.63)	0.43	2.14	(0.53, 8.62)	0.28
**Treatment-by-time point interaction**			0.74**			0.72**
Treatment vs. comparison, baseline to 3 months	0.52	(0.10, 2.77)	0.44	0.64	(0.07, 6.26)	0.70
Treatment vs. comparison, baseline to 12 months	0.67	(0.13, 3.38)	0.63	0.46	(0.06, 3.27)	0.44
**Age group (years)**						0.04**
Less than 26	–	–		Ref.		
26 to 35	–	–		0.64	(0.05, 8.37)	0.74
36 to 50	–	–		0.20	(0.02, 2.54)	0.21
51 or older	–	–		0.11	(0.01, 1.65)	0.11

*Adjusted for age category

** p-value for Wald test of interaction terms or multiple factors (e.g., multiple time points, age categories)

There was no difference in the reduction of ideation between the treatment and comparison cohorts over time. Changes in ideation were similar between cohorts from baseline to three months (OR = 0.64, 95% CI: 0.07–6.26; p = 0.70) and twelve months (OR = 0.46, 95% CI: 0.06–3.27; p = 0.44). The Wald test of the interaction between treatment and follow up indicated there is no main effect of treatment over time (p = 0.72), nor was there a secular effect of time in the comparison cohort (p = 0.71). Holding follow up time point and relevant sociodemographic factors constant, there was no difference in ideation between the treatment and comparison cohorts (OR = 2.14, 95% CI: 0.53–8.62; p = 0.28). Only age was retained in the adjusted model of main effects when evaluating sociodemographic factors for inclusion in the process outlined above. After controlling for other variables, suicidal ideation was found to decrease with increasing age category among AUD cohorts (p = 0.04).

## Discussion

We recruited 209 participants into treatment (n = 137) and comparison (n = 72) cohorts for depression, and 214 participants into treatment (n = 175) and comparison (n = 57) cohorts for AUD, within a district-wide mental healthcare program at ten health facilities in Chitwan, Nepal. The program, PRIME, provided mental health services delivered by non-specialist health workers at primary care facilities; mental health services had previously been available only at the district hospital and a private medical college. We evaluated the impact of receiving these services, which implemented WHO mhGAP guidelines for mental health care, on suicidal ideation at three and twelve months.

Our findings indicate suicidal ideation declines over the long term among people with depression, regardless of whether they receive mhGAP services. Notably, those who received mental health services from non-specialist PCWs had a significantly greater reduction in ideation over time, with this finding driven by a substantial reduction in ideation among the treatment cohort in the short term. Levels of ideation decreased over time for both treatment and comparison cohort and were similar at long-term follow up. Taken together, these results provide evidence that treatment for depression by PCWs following mhGAP guidelines benefits patients by accelerating the rate at which suicidal ideation abates, while those receiving standard health care experience a more gradual reduction over time. There is substantial value to reducing suicidality in the short term, not the least of which is a reduced likelihood of death from suicide, particularly given the high levels of reported ideation among both the treatment (48%) and comparison (43%) cohorts for depression at baseline.

Participants in the AUD cohorts reported considerably lower levels of ideation than those in the depression cohorts at baseline, with 20% of those in AUD cohorts reporting ideation compared to 45% of those in the depression cohorts. This, taken with small cohort sizes, may have led to floor effects inhibiting our ability to detect significant differences over time and between cohorts. We found no evidence mhGAP services reduce suicidal ideation among people with AUD. There were no differences in suicidal ideation over time, across treatment or comparison cohorts, or in the effect of treatment over time. The lack of impact on suicidal ideation among people with AUD is troubling given the well-established link between AUD and risk of suicide [13,17,39]. There was an inverse relationship between likelihood of ideation and age among the AUD cohorts, which is consistent with research identifying younger age groups at increased risk of suicide mortality [13].

These findings demonstrate the indirect effects of reducing clinical symptoms of depression on suicidality, rather than directly addressing suicidality as a therapeutic target. Reducing clinical symptoms and improving functionality are positive ways to affect suicidality through better overall wellbeing, and we provide evidence here to support this among people with depression. However, our evidence highlights the need for non-specialist health workers to be trained and comfortable in addressing all of a patient’s needs, including if she or he is expressing signs of suicidality. According to the depression and AUD modules of the mhGAP-IG guidelines, patients who report suicidality and pose a threat to themselves or others are identified and referred [24]. However, there are no directions for how to treat suicidality within the primary care context beyond identification within these modules. Adding the mhGAP-IG module on suicide to the roll out of the mhGAP may be an effective way to provide more direct mental health care for people at risk of suicide. Having health workers trained in this module might also augment the potential for health workers to improve suicidality among people with AUD, as well as increase the likelihood of sustained effect on reduced suicidality among people with depression, as both of these effects could not be established in the current approach. Moreover, such additional training will certainly be relevant for people who report more serious suicidal behavior, such as suicide plans or attempts.

Currently there is an insufficient evidence base to determine whether large-scale programs providing mental health services by non-specialists in primary care are an effective approach to reducing suicide in LMIC. Nepal is one of five countries within PRIME implementing a district-wide mental health care plan; research should be extended to consider the impact of these services on suicidality, particularly suicide attempts and mortality, across all country sites. Cross-cultural differences may affect the generalizability of these findings outside of Asia. Some research suggests suicide in Asia is influenced more heavily by socioeconomic circumstances than mental health issues [11], which may indicate the burden of suicide would be less affected by expanded mental health care in Nepal than in other settings. Considered another way, suicide prevention approaches in this context may need to be integrated with economic or livelihood interventions to achieve substantial reductions in mortality by suicide. Future suicide prevention programming should also address the role of gender-based violence, discrimination, and other underlying factors contributing to suicidality in South Asia [40–42].

Evidence from high-income countries on the effectiveness of large-scale mental health programs in reducing suicide mortality is also mixed. A recent study of a multi-level suicide prevention program in Europe, which included capacity building, mass media campaigns, and interventions for high-risk groups, found no impact on suicidal acts in three of four country sites [43,44]. This evidence is in line with a 2016 systematic review by Zalsman and colleagues, which found strongest evidence for restricting access to lethal means through policy interventions, followed by moderate evidence for pharmacological interventions specifically for depression, and weak evidence for family- and community-based intervention [12].

This study is not without limitations; the first of which is the limited comparability between the treatment and comparison cohorts. Participants who screened positive but did not subsequently receive a confirmatory diagnosis during diagnostic interview were enrolled into comparison conditions, which differs from a traditional control condition and limits the validity of cross-cohort comparisons. However, any bias would likely be towards the null given the comparison cohorts comprise less clinically severe samples to which the treatment cohorts are compared. Bias is nonetheless unlikely given the similar levels of reported ideation between cohorts at baseline. Secondly, we evaluate suicidal ideation rather than attempts or completions given the rare occurrence of either. While the three are all interrelated components of suicidality, we are not able to provide evidence for the impact of these services on rates of suicide attempts or mortality, only ideation. Lastly, as is typical with cohort studies, the small sample size in the comparison cohorts limits our ability to detect significant differences, particularly in subgroup analyses.

### Conclusion

Mental health services for depression provided by non-specialist primary care workers indirectly reduce suicidal ideation among this patient population in the short term. Our findings suggests the indirect benefits of treatment for depression may be in accelerating the rate at which suicidal ideation naturally abates over time. Further research is needed to understand the mechanisms through which treatment for depression reduces suicidality in this context, such as exploring the moderating effects of treatment response and adherence. Non-specialist delivered services do not appear to impact ideation among people with AUD, though findings are limited by small sample sizes, low levels of baseline ideation, and an imperfect comparison cohort. Nonetheless, these findings highlight the importance of addressing suicidality as a specific target–rather than an indirect effect–of treatment in community-based mental health care programs and support the argument for including the mhGAP module on suicidality alongside programs addressing priority mental disorders within primary care.
